# Single-operator left atrial appendage occlusion utilizing conscious sedation, transoesophageal echocardiography, lack of outpatient pre-imaging, and same-day expedited discharge: a feasibility case series

**DOI:** 10.1093/ehjcr/ytad339

**Published:** 2023-07-24

**Authors:** Hafez Golzarian, Benjamin A Pasley, Sidra R Shah, Arielle M Thiel, Gerri L Hempfling, Michael Otto, Todd Otto, Sandeep M Patel

**Affiliations:** Department of Internal Medicine, Mercy Health—St. Rita’s Medical Center, Lima, 751 West Market Street, Lima, OH 45801, USA; Department of Internal Medicine, Mercy Health—St. Rita’s Medical Center, Lima, 751 West Market Street, Lima, OH 45801, USA; Department of Internal Medicine, Mercy Health—St. Rita’s Medical Center, Lima, 751 West Market Street, Lima, OH 45801, USA; Structural Heart and Intervention Center, Mercy Health—St. Rita’s Medical Center, 730 West Market Street, 2K Tower, Lima, OH 45801, USA; Structural Heart and Intervention Center, Mercy Health—St. Rita’s Medical Center, 730 West Market Street, 2K Tower, Lima, OH 45801, USA; Structural Heart and Intervention Center, Mercy Health—St. Rita’s Medical Center, 730 West Market Street, 2K Tower, Lima, OH 45801, USA; Structural Heart and Intervention Center, Mercy Health—St. Rita’s Medical Center, 730 West Market Street, 2K Tower, Lima, OH 45801, USA; Structural Heart and Intervention Center, Mercy Health—St. Rita’s Medical Center, 730 West Market Street, 2K Tower, Lima, OH 45801, USA

**Keywords:** WATCHMAN, SOLO-CLOSE, Single operator, Conscious sedation, Early discharge, Left atrial appendage occlusion, Case series, TEE

## Abstract

**Background:**

Contemporary procedural guidelines for percutaneous left atrial appendage occlusions (LAAO) with the WATCHMAN device often require the utilization of pre-screening imaging, general anaesthesia, intubation, a dedicated intra-procedural echocardiographer, and overnight observation. For these reasons, LAAO with the WATCHMAN is not economically feasible for many hospital systems. Thus, we sought to evaluate a newstrategy for implantation that may provide a more minimalistic and less cumbersome approach to LAAO.

**Case summary:**

We describe five cases utilizing single-operator left atrial appendage occlusion utilizing conscious sedation, transoesophageal echocardiography, lack of outpatient pre-imaging, and same-day expedited discharge (SOLO-CLOSE)—a novel single-operator procedural strategy for LAAO that safely foregoes the aforementioned procedural requirements and allows for same-day early discharge. All five patients were observed according to our newly devised SOLO-CLOSE protocol and were safely discharged home the same day. Follow-up transoesophageal echocardiography (TEE) at 45 days and 1 year revealed well-seated and well-anchored devices with no leaks (<5 mm) or device-related thrombi.

**Discussion:**

The SOLO-CLOSE series is the first ever documented WATCHMAN strategy that utilizes a single-operator, TEE-guided, nurse-driven conscious sedation protocol that defers pre-screening imaging and allows for same-day discharge. The versatility of this technique allows proceduralists to comfortably achieve successful LAAO despite a wide range of risk profiles. This single-operator technique has potential to become a widely accepted universal approach for non-pharmacological cardioembolic stroke prophylaxis due to its efficacy, safety, simplicity, and presumable cost-effectiveness.

**Learning points**

- Single-operator left atrial appendage occlusion utilizing conscious sedation, transoesophageal echocardiography, lack of outpatient pre-imaging, and same-day expedited discharge (SOLO-CLOSE) is an efficacious strategy for left atrial appendage occlusion that utilizes a single-operator and nurse-driven conscious sedation protocol that allows for safe and cost-effective outcomes.
- SOLO-CLOSE has potential to become a widely accepted approach for non-pharmacological cardioembolic stroke prophylaxis for structural heart centres around the world.

## Introduction

Percutaneous left atrial appendage occlusion (LAAO) with the WATCHMAN FLX device is a non-pharmacologic alternative for cardioembolic stroke prophylaxis in patients with atrial fibrillation. In the USA, LAAO is generally performed under general anaesthesia and pre-procedural echocardiographic guidance. The newest iteration—WATCHMAN FLX—has improved ease of deployment, manoeuvrability, and recapturability. Additionally, it met the primary effectiveness end point with a left atrial appendage closure incidence of 100.0% at both 45 days and 1 year.^1^ Despite device enhancements, procedural methodology has not followed suit and remains unchanged. Contemporary approaches of LAAO with the WATCHMAN are not economically feasible for many hospital systems as they frequently require the use of pre-screening imaging, general anaesthesia, intubation, an intra-procedural echocardiographer, and overnight inpatient admission. Similar to the transcatheter aortic valve replacement evolution, we demonstrate a previously undescribed strategy for percutaneous LAAO with WATCHMAN FLX using a single-operator and nurse-driven conscious sedation protocol that defers pre-screening imaging and allows for early same-day discharge. We refer to this minimalist LAAO approach as single-operator left atrial appendage occlusion utilizing conscious sedation, transoesophageal echocardiography, lack of outpatient pre-imaging, and same-day expedited discharge (SOLO-CLOSE). We describe five SOLO-CLOSE cases and discuss why it is an appealing percutaneous LAAO strategy for structural heart programmes around the world.

## Summary figure

**Table:** 

**00:00:00:**	Patient brought to the procedural suite.
**00:01:32:**	Patient placed on cardiac monitors.
**00:01:56:**	Non-rebreather mask and bite block applied.
**00:02:39:**	Right groin cleaned and prepped, draped in sterile fashion.
**00:03:55:**	Physician scrubbed in.
**00:04:09:**	Procedure time-out completed and documented.
**00:04:29:**	Nurse-driven conscious sedation protocol initiated.
**00:04:44:**	Transoesophageal echocardiography (TEE) probe inserted by a single operator.
**00:14:44:**	Left atrial appendage anatomical assessment and device sizing completed.
**00:15:02:**	Probe held in place with wheel-lock mechanism, clips, and towels. X-plane imaging initiated.
**00:17:04:**	Ultrasound-guided right femoral vein access was performed. Sheath inserted.
**00:18:51:**	2000-unit heparin administered.
**00:20:54:**	The septum was successfully crossed.
**00:31:25:**	WATCHMAN FLX device was deployed.
**00:31:37:**	TEE resumed for post-deployment assessment.
**00:34:32:**	PASS criteria met. Procedure complete. All equipment removed.
**00:36:04:**	The access site was closed with a closure device; supplemental manual pressure applied by staff.
**00:36:10:**	Physician scrubs out. Family updated.
**00:46:43:**	Dressing applied to access site by staff. The patient was transferred to the recovery unit. Vital signs were assessed.
**02:47:43:**	Access site was assessed for any bleeding or oozing.
**04:33:23:**	Patient was prompted to ambulate and work with physical therapy.
**04:37:23:**	Ambulation was successful. Vital signs assessed.
**05:05:23:**	Pre-discharge TTE performed.
**05:14:05:**	TTE complete.
**05:42:00:**	Patient provided with discharge instructions and discharged home with next-day follow-up scheduled.
**20:34:00:**	Patient presents to clinic for follow-up. Access site was re-assessed. 45-day TEE scheduled.

## Methods

The novelty of the SOLO-CLOSE strategy lays in its requirement of only a single operator to complete the LAAO. Patients with non-valvular atrial fibrillation who wish to undergo the WATCHMAN procedure and are screened appropriately may qualify for the SOLO-CLOSE protocol. Specific deferral criteria are summarized in *Table 1*. We describe the typical procedural methodology. The right femoral vein is chosen as the primary point of access. A procedural oxygen mask with an orifice that allows for the insertion of a transoesophageal echocardiography (TEE) probe while delivering high-flow oxygen is applied to the patient. Nurse-driven conscious sedation protocol is then initiated. A covered TEE probe is then inserted via the perforated orifice to allow for sterile manipulation during the procedure by the operator. Upon completion of anatomical assessment of the left atrial appendage (LAA), the probe is then secured in the bicaval view using wheel-lock mechanisms, towels, and clips. X-plane echocardiography is initiated simultaneously. Thereafter, a standard 12-French sheath is inserted into the right femoral vein under ultrasound guidance. Through this sheath, a 0.035 guide wire is inserted into the superior vena cava, and over this wire, an 8.5-French pre-shaped Baylis VersaCross sheath is introduced. We then exchange out for the Baylis radiofrequency wire and perform a typical inferoposterior trans-septal puncture and advance the Baylis wire into the left atrium followed by the VersaCross sheath. The femoral venous 12-French and VersaCross sheaths are removed over the Baylis wire, and a standard WATCHMAN delivery sheath (typically double-curve) is then inserted in sheathless fashion into the left atrium (LA). A 5-French pigtail catheter is then inserted into the WATCHMAN delivery sheath and manipulated into the LAA. In the right anterior oblique caudal projection with concomitant TEE, angiography of the appendage is performed. Based on this and the intra-procedural imaging, the size of the WATCHMAN FLX device is determined. The selected device is then prepared per manufacturer’s recommendations, appropriately deaired, and then positioned using fluoroscopy and the 135-degree view on TEE. We then unsheathe the device slowly and, using the FLX ball technique, ensure that the position of the device is appropriate before releasing the shoulders of the device. We then perform our usual post-deployment assessment including a tug test, and then once position, anchor, size, and seal criteria (PASS) are assessed and met, the device is finally released. All equipment is then removed, and a modified figure-of-eight suture is used for haemostasis. Transoesophageal echocardiography is performed again prior to procedural completion to ensure no pericardial effusion is seen, the device is assessed once more for stability and final position, and lastly, the inter-atrial septum is evaluated for the shunt and its haemodynamic effects to assess for the need for closure. The patient is monitored over the next 4–6 h to determine candidacy of same-day discharge. *Table 2* summarizes our criteria for early discharge. Transoesophageal echocardiography is performed immediately prior to discharge. Patients are provided with an emergency department card as well as our structural heart office number in case any complication(s) arise(s). A follow-up phone call is done the next day, and if needed, a clinic visit is scheduled; otherwise, the patient follows up at 45 days for clinical evaluation and repeat TEE.

**Table 1:** Suggested SOLO-CLOSE deferral screening criteria

**Allergy to anaesthetic components**
**ASA Class ≥ 4^a^**
**Combative nature of patient with anaesthetics**
**Oesophageal strictures or varices**
**History of anaesthesia-related complications**
**History of difficult endotracheal intubation**
**Inability to lay flat for prolonged periods**
**Mallampati Class 4**
**Moderate dementia or worse**
**Morbid obesity**
**Severe chronic obstructive pulmonary disease**
**Small posterior pharynx**

a ASA classes are defined as the patient physical status classifications developed by the American Society of Anesthesiologists (ASA) to help predict operative risk. The classes range from 1 to 6. We define ASA Class 4 as patients with severe systemic disease that is poorly controlled and life-threatening (i.e. decompensated heart failure, chronic obstructive pulmonary disease, and unstable angina).^2^

**Table 2:** Suggested SOLO-CLOSE early discharge criteria

**Uncomplicated use of conscious sedation**
**Uncomplicated vascular access and closure**
**Uncomplicated trans-septal puncture**
**Post-procedural haemodynamic stability**
**Absent intra-procedural pericardial effusion**
**Patient willingness to go home with a caregiver**
**Patient transportation availability**
**Able to tolerate oral intake**
**Able to ambulate at baseline 4–6 h after the procedure**
**Tolerating oral anticoagulation post**-**procedure**
**Absent cardiac symptoms at time of discharge**
**Post-procedural transthoracic echocardiogram without abnormalities**

## Case presentations

### Patient 1: conscious sedation using dexmedetomidine with supplemental fentanyl and midazolam

**Figure 1:**
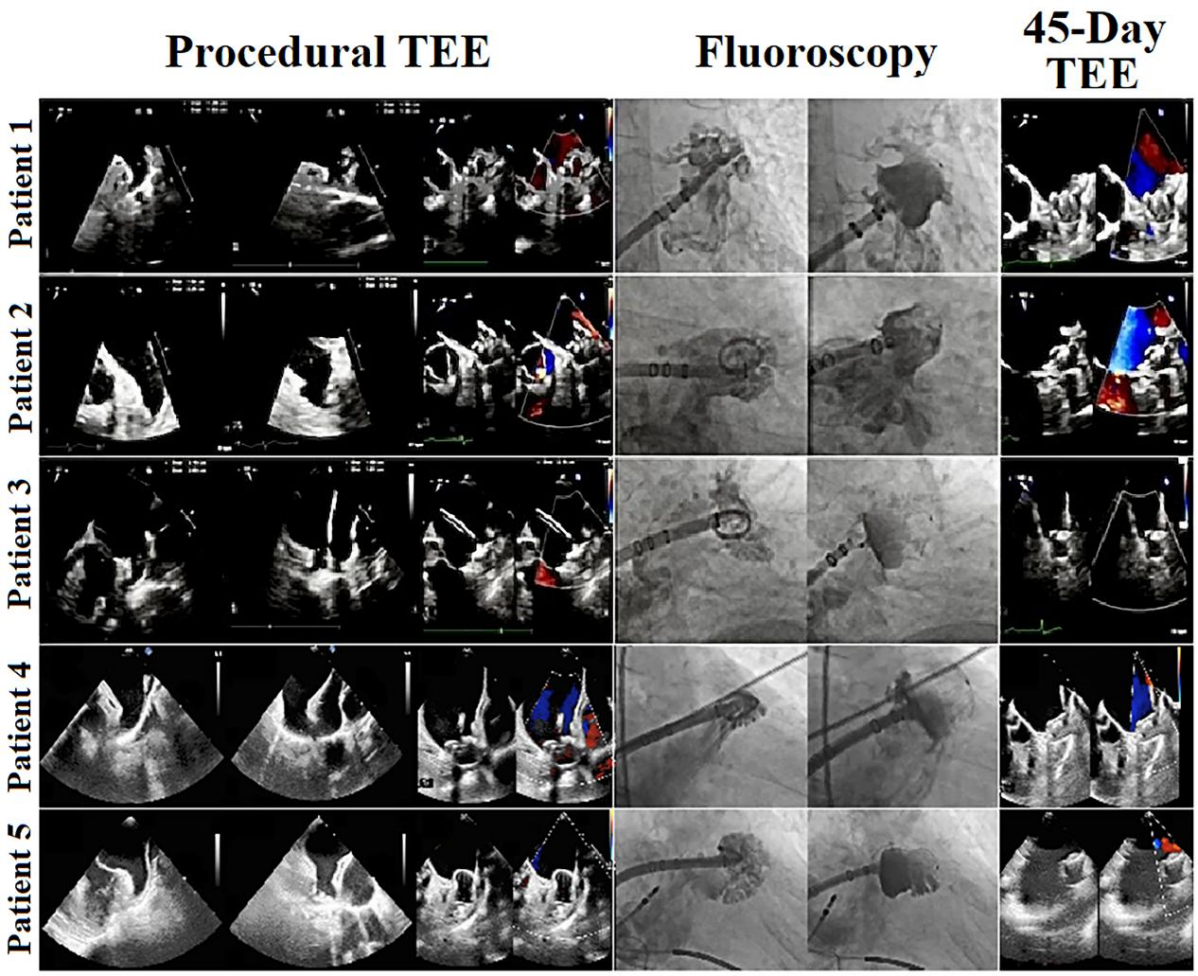
Successful implantations of WATCHMAN FLX devices using the SOLO-CLOSE strategy with 45-day postoperative transoesophageal echocardiography revealing complete sealing with no peri-valvular leaks. TEE, transoesophageal echocardiography.

An 81-year-old female with history of paroxysmal atrial fibrillation on warfarin (CHADS2VASC = 6, HASBLED = 5), transient ischaemic attack, and hypertension developed gastrointestinal haemorrhage related to ulcerations and elevated international normalized ratio. Due to her frailty, the structural heart team elected a SOLO-CLOSE approach. Low-dose intravenous (IV) dexmedetomidine was initiated prior to the procedure with boluses of IV fentanyl and midazolam available as needed. Using the SOLO-CLOSE technique, a WATCHMAN FLX 20-mm device was successfully deployed and released (*Figure 1*).

### Patient 2: conscious sedation using propofol with supplemental fentanyl and midazolam

A 73-year-old male with history of hypertension, non-obstructive coronary artery disease, and paroxysmal atrial fibrillation (CHADS2VASC = 3, HASBLED = 3) presented for LAAO evaluation due to episodes of rectal bleeding. Due to a history of post-anaesthesia confusion, the patient was deemed appropriate for SOLO-CLOSE. Intravenous propofol bolus was administered and a SOLO-CLOSE technique ensued. Small aliquots of propofol, fentanyl, and midazolam were used as needed. Intra-procedural appendage measurements were appropriate for a WATCHMAN FLX 24-mm device, which was deployed and released with no complication (*Figure 1*).

### Patient 3: conscious sedation using fentanyl and midazolam only

An 81-year-old male with history of diabetes mellitus, gastric ulcers, transient ischaemic attack, chronic diastolic heart failure, non-obstructive coronary artery disease, chronic hypotension, recurrent falls, and long-standing persistent atrial fibrillation (CHADS2VASC = 7, HASBLED = 4) presented for LAAO. Due to co-morbidities, a SOLO-CLOSE approach was elected. Intravenous fentanyl and midazolam were used for sedation. Based on intra-procedural measurements, it was determined that a WATCHMAN FLX 27 mm would be appropriate; this was ultimately deployed without complication (*Figure 1*).

### Patient 4: conscious sedation using dexmedetomidine with supplemental fentanyl and midazolam

A 90-year-old female with history of chronic diastolic heart failure, hypertension, hyperlipidaemia, colon cancer status post-colectomy, and permanent atrial fibrillation (CHADS2VASC = 5, HASBLED = 4) on dabigatran with recurrent gastrointestinal bleeds presented for LAAO. The SOLO-CLOSE approach was elected. Low-dose IV dexmedetomidine was initiated prior to the procedure with boluses of IV fentanyl and midazolam available as needed. A WATCHMAN FLX 27 mm was deployed without difficulty (*Figure 1*).

### Patient 5: conscious sedation with dexmedetomidine with supplemental midazolam only

A 71-year-old female with history of end-stage renal disease, prior haemorrhagic stroke, hypertension, diabetes mellitus, hyperlipidaemia, chronic diastolic heart failure, and long-standing persistent atrial fibrillation (CHADS2VASC = 8, HASBLED = 6) presented for LAAO. Due to co-morbidities, the SOLO-CLOSE methodologic was utilized. Low-dose IV dexmedetomidine was initiated prior to the procedure with boluses of midazolam available as needed. Oral hydromorphone was administered pre- and post-procedurally. Transoesophageal echocardiography determined that a WATCHMAN FLX 27 mm would be appropriate; this was ultimately deployed without complication (*Figure 1*).

### Results

All five patients were observed according to protocol (*Table 2*). They were subsequently safely discharged home on antiplatelet therapy per standard guidelines. Forty-five-day follow-up TEE revealed well-seated and well-anchored devices, with no peri-device leaks (<5mm) or any device-related thrombi (*Figure 1*).

## Discussion

Percutaneous LAAO with the WATCHMAN has become an appealing option for stroke prophylaxis in patients with atrial fibrillation and elevated stroke risk in the setting of contraindications to long-term oral anticoagulation.^3^ The conventional protocol for WATCHMAN implantation necessitates pre-screening imaging, endotracheal intubation, general anaesthesia, intra-procedural echocardiography, and inpatient monitoring overnight. Our case series is the first contemporary description of a modified approach to all of these aspects of the WATCHMAN procedure.

Screening imaging with TEE or computed tomographic angiography prior to the procedure day has been recommended to ensure appropriateness for LAAO when using the Watchman device.^4^ We argue that this may not be necessary with the WATCHMAN FLX device. The WATCHMAN FLX has a size matrix that is able to effectively seal greater than 98% of the population. It is a much more versatile device that allows for ease in manipulation once in the appendage using the ‘FLX ball technique’. In fact, 100% of patients demonstrated effective appendage closure at 1-year follow-up in the PINNACLE FLX trial, irrespective of anatomy.^1^ Thus, we suggest echocardiography done on the day of the procedure in the procedural suite. This appears to be cost-effective and avoids the complexity of an additional pre-procedure test.

Suggested minimum competency standards that individual team members should achieve to obtain SOLO-CLOSE proficiency are outlined in *Table 3*. The primary criticisms of single-operator TEE are two-fold: the need for prolonged deep sedation to avoid patient motion while preserving respiratory function and the ability of the proceduralist to operate the TEE probe while performing the procedure. To address the first issue, our approach was to build upon pre-existing evidence that various anaesthetics have been shown to be effective at achieving adequate sedation and anxiolysis to allow probe manipulation. As demonstrated in our case series, the use of dexmedetomidine, low-dose propofol, and fentanyl with midazolam provided adequate anaesthesia without concerns for respiratory depression. These agents have previously been used for TEE-based procedures and have consistently provided safe and effective analgesia.^5–9^ In fact, conscious sedation protocols for WATCHMAN have demonstrated significant efficacy outside of the USA.^10^*Table 4* summarizes the various combinations of anaesthetics our team utilized for conscious sedation to promote safe early discharge. We utilized these various combinations to demonstrate the feasibility of performing LAAO with WATCHMAN under conscious sedation regardless of the type of anaesthetic medications utilized.

**Table 3:** Suggested structural heart team-based competencies to achieve SOLO-CLOSE proficiency

**Anaesthesiologist**	To determine whether the patient is a candidate for conscious sedation based on history and airway anatomy prior to procedure
**Echocardiographic technician**	Proficiency with rapid 3D and multi-planar imaging with measurements
**Procedural nurse**	To be able to manage IV sedatives and reversal agents
	To be able to initiate positive airway ventilation when needed
**Proceduralist**	To be able to perform and interpret TEE from the right side of the patient
	To be able to perform percutaneous LAAO while directing procedural sedation and manipulating the TEE probe
**Proceduralist scrub technician**	To provide minimal support or stabilization of the TEE probe (no major manipulation)
**Post-procedure nurse**	Access site management
	Reversal agent competency
	To provide patient with education and discharge instructions
**Structural heart coordinator**	To identify patients who meet specific criteria for SOLO-CLOSE
	To establish expectations with patients and families
	To coordinate same-day discharge planning and follow-up

IV, intravenous; LAAO, left atrial appendage occlusion; TEE, transoesophageal echocardiography.

**Table 4:** Data regarding the various combinations of anaesthesia used for conscious sedation

Patient	CHA_2_DS_2_VASc; HAS-BLED	Concurrent dexmedetomidine	Fentanyl administration	Midazolam administration	Propofol administration	WATCHMAN FLX device	Procedure time	Same-day discharge
**1**	6; 5	Yes	50 mcg	2.0 mg	0 mg/kg	20 mm	30 min	Yes
**2**	3; 3	No	125 mcg	3.0 mg	1.2 mg/kg	24 mm	26 min	Yes
**3**	7; 4	No	25 mcg	0.5 mg	0 mg/kg	27 mm	25 min	Yes
**4**	5; 4	Yes	50 mcg	1.0 mg	0 mg/kg	27 mm	29 min	Yes
**5**	8; 6	Yes	0 mcg	2.0 mg	0 mg/kg	27 mm	20 min	Yes

Recorded procedure time is defined as the time from insertion of the TEE probe until the time of WATCHMAN FLX device deployment by the single operator.

With respect to the concerns of performing single-operator TEE, we would recommend that the operator focuses on performing non-LAAO TEE cases from the right side of the patient, in a position commensurate to where the proceduralist would stand when performing the WATCHMAN procedure. The learning curve of performing TEE from this position will be time-consuming initially but will help familiarize the operator with the various adjustments in probe manipulation necessary to be able to produce adequate imaging during LAAO. Once this hurdle is crossed, we hypothesize that the single-operator TEE will no longer be a hindrance but will become an asset due to the increased level of control afforded to the proceduralist. Another major benefit to a single-operator approach that is gaining traction in recent literature is the elimination of unnecessary radiation exposure to intra-procedural echocardiographers.^11^

Contemporary proceduralists are invoking an alternative method of simplifying LAAO by utilizing intra-cardiac echocardiography (ICE).^12^ Three-dimensional ICE provides the option to forgo intra-procedural TEE (and general anaesthesia), thus theoretically also improving efficiency and reducing costs. Despite perceived advantages of ICE, there are clear topics of concern: the need for a second venous access, the possibility of a second trans-septal puncture, an increase in thrombotic risk with an additional intra-cardiac device, the need for catheter manipulations in the pulmonary artery/left atrium (perforation risk), non-standard imaging planes, and the lack of standardized criteria for procedural guidance, device assessment, and release. Additionally, ICE imaging requires a greater capital investment in terms of the console, imaging software, and disposable imaging catheters. When considering the aforementioned issues, the use of ICE for LAAO may in fact paradoxically have a negative effect on procedural efficiency and economics.

Lastly, a cornerstone to the SOLO-CLOSE strategy is same-day discharge. Our criteria for qualifying for same-day discharge included an uncomplicated procedure, use of vascular haemostasis devices, a minimum of 4 h of an uneventful observation period, baseline oxygenation and ambulation status, completion of a limited pre-discharge TTE demonstrating no peri-device leakage, and patient agreement to next-day follow-up if needed (*Table 2*). Prior to discharge, our patients are provided with an emergency contact card linked to our emergency department and structural heart clinic which patients may utilize should any post-procedural complications arise. Otherwise, a follow-up nurse phone call is made the following day and a clinical visit is scheduled only if needed. All patients then undergo follow-up with TEE at 45 days and 1 year per standard protocol. As many transcatheter procedures were also once deemed complex procedures requiring overnight admission (percutaneous coronary intervention, atrial septal defect closure, atrial fibrillation ablation, and in some instances transcatheter aortic valve replacement), we anticipate that LAAO will similarly also evolve and follow suit. Successful evolution requires large-scale studies to develop and validate clearly delineated criteria for same-day discharge candidacy, similar to that which we have attempted at our institution (*Table 2*). Recent and ongoing studies reveal no difference in safety or efficacy of LAAO between same-day and next-day discharge patients.^13–16^ Furthermore, this approach has potential to significantly reduce resource utilization and costs while improving patient and provider satisfaction.

As with any procedure, single operators utilizing SOLO-CLOSE should be trained and equipped to identify and manage any potential procedural complications which include but are not limited to respiratory depression and cardiac tamponade. Conscious sedation should be monitored by the proceduralist and nursing personnel that are appropriately trained in managing supplemental oxygen delivery and providing appropriate antidotes. Further, LAAO often requires the operator to perform a posterior and inferior transeptal puncture to have a coaxial trajectory to the main body of the LAA. Although rare, this may lead to accidental puncture of the muscular portion of the septum which can lead to a delayed tamponade or effusion. In many such instances, single operators have the immediate ability to alter their own imaging to evaluate the pericardial space and expeditiously guide therapeutic pericardiocentesis.^17^

### Limitations

The SOLO-CLOSE protocol has not been studied extensively and is our group’s attempt at evolving the methodology of LAAO. Due to its inherent learning curve, procedure times for the SOLO-CLOSE may initially be prolonged but are expected to significantly improve with proficiency and will allow for synchronization between device manipulation and echocardiographic intra-procedural imaging. We anticipate that the SOLO-CLOSE methodology will significantly reduce healthcare costs. However, large-scale prospective observational studies are needed to further assess the efficacy and safety of this approach.

## Conclusion

Our series is the first documented WATCHMAN strategy that utilizes a single-operator, TEE-guided, nurse-driven conscious sedation protocol that defers pre-screening imaging and prioritizes same-day discharge. The versatility and ubiquitous applicability of this technique allows proceduralists to comfortably achieve successful LAAO despite a wide range of risk profiles. We believe SOLO-CLOSE has potential to become a widely accepted approach for non-pharmacological cardioembolic stroke prophylaxis due to its efficacy, safety, simplicity, and potential for cost-effectiveness.

## Data Availability

The data that support the findings of this study are available from the authors upon request.

## Lead author biography

Dr Sandeep M. Patel MD, FACC, FSCAI, RPVI, is the medical director of the Structural Heart & Intervention Center at Mercy Health—St. Rita’s Medical Center in Ohio, USA. He has experience in performing complex coronary, peripheral, and structural heart interventions. He has a specific interest in delivering high-quality care in a cost-effective manner. He is the pioneer of the SOLO-CLOSE methodology for LAAO which is rapidly gaining popularity amongst many structural heart centres around the world.
